# Immunosuppression through HNF-1β signaling in human ovarian clear cell cancer

**DOI:** 10.1186/2051-1426-1-S1-P168

**Published:** 2013-11-07

**Authors:** Hiroshi Nishio, Juri Sugiyama, Tomonori Yaguchi, Daisuke Aoki, Yutaka Kawakami

**Affiliations:** 1 Division of Cellular Signaling, Advanced Medical and Science Research, School of Medicine, Keio University, Tokyo, Japan; 2 Obstetrics and Gynecology, School of Medicine, Keio University, Shinjuku-ku, Japan

## 

Cancer-induced immunosuppression is one of the major problems for development of cancer immunotherapies. A transcriptional factor HNF-1β preferentially activated in human ovarian clear cell cancer (OCCC) was reported to contribute to various malignant features including metastases and glucose metabolism. In this study, we have investigated roles of HNF-1β in the immunosuppressive activity of human OCCC. HNF-1β knockdown and overexpression experiments revealed that HNF-1β induced production of IL6 and IL8, which were elevated in OCCC patient plasma. HNF-1β was found to promote production of IL6 and IL8 through activated STAT3 signaling and NF-κB dependent osteopontin pathway. In vitro suppressive activities of human OCCC culture supernatants on generation of human monocyte-derived dendritic cell (DC) were reduced by siRNA knockdown of HNF-1β in cancer cells partly through decrease of IL6 production. In the nude mice implanted with human OCCC cell lines, knockdown of HNF-1β in the cancer cells resulted in restoration of T cell stimulatory activity of murine splenic DC, and decrease of accumulation and arginase expression of myeloid-derived suppressor cells in spleens and tumors accompanied by human IL6 decrease. In the OCCC patient plasma, IL8 levels were correlated with the levels of immunosuppressive arginase, indicating that IL8 may also be involved in immunosuppression. Therefore, HNF-1β activation in human OCCC is an upstream event for induction of immunosuppression via STAT3 and NF-κB activation, and is an attractive target for restoring immunocompetence in OCCC patients.

